# Monitoring of diffusion properties and transverse relaxation time of mouse ischaemic muscle after administration of human mesenchymal stromal cells derived from adipose tissue

**DOI:** 10.1111/cpr.12672

**Published:** 2019-08-23

**Authors:** Agnieszka Skorupa, Mateusz Ciszek, Ewelina Pilny, Ryszard Smolarczyk, Magdalena Jarosz–Biej, Łukasz Boguszewicz, Łukasz Krakowczyk, Stanisław Szala, Maria Sokół, Tomasz Cichoń

**Affiliations:** ^1^ Department of Medical Physics Maria Sklodowska–Curie Institute - Oncology Center, Gliwice Branch Gliwice Poland; ^2^ Center for Translational Research and Molecular Biology of Cancer Maria Skłodowska-Curie Institute - Oncology Center, Gliwice Branch Gliwice Poland; ^3^ Department of Oncologic and Reconstructive Surgery Maria Sklodowska-Curie Institute - Oncology Center, Gliwice Branch Gliwice Poland

**Keywords:** diffusion, ischaemia, magnetic resonance imaging, mesenchymal stromal cells, peripheral artery disease, transplantation

## Abstract

**Objectives:**

Application of non‐invasive imaging methods plays an important role in the assessment of cellular therapy effects in peripheral artery disease. The purpose of this work was to evaluate the kinetics of MRI‐derived parameters characterizing ischaemic hindlimb muscle after administration of human mesenchymal stromal cells derived from adipose tissue (hADSC) in mice.

**Materials and methods:**

MRI experiments were performed on a 9.4T Bruker system. The measurement protocol included transverse relaxation time mapping and diffusion tensor imaging. The monitoring period encompassed 14 days after femoral artery ligation and subsequent cell administration. The effect of hADSC transplantation was compared with the effect of normal human dermal fibroblasts (NHDFs) and phosphate‐buffered saline injection.

**Results:**

The most significant differences between the hADSC group and the remaining ones were observed around day 3 after ischaemia induction (increased transverse relaxation time in the hADSC group in comparison with the control group) and around day 7 (increased transverse relaxation time and decreased third eigenvalue of the diffusion tensor in the hADSC group in comparison with the control and NHDF groups) at the site of hADSC injection. Histologically, it was associated with increased macrophage infiltration at days 3‐7 and with the presence of small regenerating fibres in the ischaemic tissue at day 7.

**Conclusions:**

Our results underscore the important role of macrophages in mediating the therapeutic effects of hADSCs and confirm the huge potential of magnetic resonance imaging in monitoring of cellular therapy effects.

## 1 INTRODUCTION

From 3% to 10% of the population in the Western world suffer from peripheral arterial disease, and this represents a major health problem of ageing population.

The natural response to major artery occlusion is a complex process, which can be divided into three phases (with some overlapping): (a) cell necrosis and apoptosis phase caused by hypoxia; (b) inflammatory phase characterized by lymphocyte, monocyte and neutrophil migration into the damaged tissue for removal of dead cells; and (c) the muscle regeneration phase.^1^ During this process, vasculogenesis, angiogenesis and arteriogenesis occur to establish a functional vascular network. In appropriate conditions, skeletal muscle has the ability to recover after ischaemic injury. However, post‐ischaemic vessel growth and remodelling processes are markedly impaired in patients suffering from arterial diseases. Critical limb ischaemia—an advanced stage of this disease—is associated with a high risk of amputation and death.^2^ The prevalence of this condition is approximately 1.3%.^3^ Therefore, new therapeutic approaches for these patients are being searched.

Application of mesenchymal stromal cells (MSCs) in the repair of ischaemic tissues has been extensively studied.^4^, ^5^, ^6^ MSCs are multipotent (differentiating into adipocytes, chondrocytes and osteoblasts), non‐haematopoietic, fibroblast‐like plastic adherent cells that can be isolated from various tissue sources (including bone marrow and adipose tissue). Their therapeutic potential is mainly related to the secretion of growth factors and interleukins that can have immunomodulatory, angiogenic, anti‐inflammatory and anti‐apoptotic effects (the paracrine effect). They may also be a vehicle for gene therapy and drug delivery. Although the initial enthusiasm that these cells are immune‐privileged has diminished, it is accepted that they are less immunogenic than other cell types.^7^ This property encourages researchers to exploit human rather than mouse MSCs in the studies of mouse models of various diseases.^8^ Such studies provide useful preclinical data necessary for designing of clinical trials. Importantly, human MSCs are easier to isolate, expand in culture and less prone to undergo spontaneous transformation to tumorigenic cells than mouse MSCs. A large number of works indicate that human MSCs may exert immunosuppressive effect in immunocompetent mice.^8^, ^9^, ^10^ On the other hand, macrophage infiltration, suggestive of transplant rejection, was also observed after administration of human MSCs to rodents.^11^, ^12^ Huge variability of the results can be partly explained by the plasticity of MSC phenotype depending on the microenvironment of the host.^13^, ^14^

The research progress in the field of cellular therapy depends substantially on the application of non‐invasive imaging methods allowing in vivo monitoring of the effects of therapeutic interventions. Magnetic resonance imaging (MRI) technique offering several contrast mechanisms is well suited for a multiparametric characterization of injured tissue. The transverse relaxation time (T2) is known to increase with tissue oedema, necrosis and inflammation.^15^, ^16^ Due to fibrillar muscle structure, self‐diffusion of water is restricted by membranes and is greater along the fibre orientation than in other directions. Diffusion‐weighted magnetic resonance imaging (DWI or DW‐MRI) and its special kind, diffusion tensor imaging (DTI), allow the mapping of the diffusion process of water.^17^ Diffusion tensor is usually calculated from six or more different diffusion‐weighted acquisitions, each obtained with a different orientation of the diffusion‐sensitizing gradients. The first eigenvector of this tensor describes the fibre direction, while the second and third eigenvectors represent diffusion perpendicular to the long axis of the cell. The corresponding eigenvalues (*λ*_1_, *λ*_2_ and *λ*_3_) are the diffusion coefficients along these directions, while fractional anisotropy (FA) describes the degree of anisotropy of diffusion. Therefore, quantitative indexes obtained from diffusion tensor imaging characterize local tissue microarchitecture.

The parameters obtained from relaxation and diffusion imaging of ischaemic hindlimbs were found to change dynamically during degeneration and regeneration processes after femoral artery ligation 18, 19, 20, 21 and provided a useful insight into the therapeutic effects of human and mouse endothelial progenitor cells in mouse models of hindlimb ischaemia.^22^, ^23^

Recently, our group has reported that M2 macrophages are involved in the repair process of ischaemic muscle tissue after transplantation of hADSC in immunocompetent mice.^24^ The purpose of this work was to evaluate the changes in diffusion and relaxation properties of muscle tissue during this process.

## 2 MATERIALS AND METHODS

### 2.1 Ethical statement

The experiments were performed in accordance with the Declaration of Helsinki, with the approval of the Local Committee on Bioethics in Katowice. The experimental protocol was approved by the Local Ethics Commission (KB430‐17/14).

### 2.2 Isolation of hADSCs

Mesenchymal stromal cells were isolated from subcutaneous adipose tissues collected during surgery in Maria Skłodowska‐Curie Institute ‐ Oncology Center, Gliwice Branch (Poland). The adipose tissues were digested with collagenase using a modification of a previously described protocol (according to Rossini et al^25^).

### 2.3 Cell culture

Cells were cultured in DMEM with high glucose, supplemented with 10% (NHDF [Lonza]) or 20% (ADSC) FBS and antibiotics (penicillin and streptomycin).

### 2.4 Mouse model of hindlimb ischaemia

In our work, we used the commonly exploited femoral artery ligation model. 10‐ to 12‐week‐old male C57BL/6NCrl (immunocompetent) mice were anaesthetized with 2% isoflurane and underwent surgical ligation of the left femoral artery to create unilateral hindlimb ischaemia. The artery was ligated at two points using surgical sutures, according to Brenes et al^26^ This step was not followed by artery excision.

We are aware that variation in surgical procedures (femoral artery ligation vs ligation + excision of all branches) leads to the variability in the level of ischaemia. Since our study was devoted to the assessment of M2 macrophage role in the repair of ischaemic tissue, we wanted to limit the surgery‐related tissue damage. Although vessel excision would result in more severe ischaemia, diffusion tensor imaging and relaxometry have already been shown efficient in visualization of degeneration and regeneration processes after femoral artery ligation in mice (Heemskerk et al).^18^ Therefore, we aimed to evaluate the influence of hADSC administration on these processes.

Although we expected to observe ischaemic changes across the entire calf muscle cross‐section, the therapeutic cells were administered into the gastrocnemius muscle (posterior ROI; see Section 2.72.2), according to Brenes et al.^26^

### 2.5 Administration of hADSCs and NHDF cells

An hour after ligation, 1 × 10^6^ hADSC cells and 1 × 10^6^ NHDF cells in 100 μL of PBS were administered into the gastrocnemius muscles of the mice. The control mice were injected with 100 μL of PBS. The total number of mice included in the study was 57 (19 per each group).

### 2.6 Magnetic resonance imaging

Magnetic resonance imaging experiments were performed on a 9.4 T vertical 89 mm bore system (Bruker BioSpin) equipped with a Bruker Micro 2.5 gradient system and a transmit/receive birdcage radiofrequency coil with an inner diameter of 30 mm. During data acquisition, the animals were anaesthetized with 2%‐3% sevoflurane. Body temperature and respiration rate were monitored using ECG/respiratory unit (SA Instruments, Inc). A field‐map–based shimming (MAPSHIM, paravision 6.0) was used to optimize B0 field homogeneity.

Effectiveness of femoral artery ligation was visually evaluated using magnetic resonance angiography. The images of vascular system were acquired using a multislice 2D TOF (time of flight) flow‐compensated sequence using the following parameters: echo time = 3.1 ms, repetition time = 12 ms, flip angle = 80º, 2 cm × 3 cm field of view, 200 × 300 matrix size, 0.3 mm slice thickness, 0.2 mm inter‐slice distance and three signal averages.

The imaging protocol included the following:

- T2‐weighted RARE image sequence (repetition time = 2500 ms, echo time = 20 ms, RARE factor = 4, flip angle = 90°, 3 cm × 2 cm field of view, 300 × 160 matrix size, 1.5 mm slice thickness, 0.5 mm gap between slices, 10 slices and 2 signal averages);
- Diffusion‐weighted spin echo sequence with fat suppression (diffusion gradients applied in 12 non‐collinear directions and a reference image acquired without diffusion weighting, repetition time = 1300 ms, echo time = 28 ms, diffusion gradient duration of 7 ms, diffusion gradient separation time of 14 ms, b value of 500 s/mm^2^, 3 cm × 2 cm field of view, 180 × 60 matrix size, 1.5 mm slice thickness, 10 slices, 0.5 mm gap between slices and two signal averages);
- Multislice multiecho sequence with fat suppression (repetition time = 4000 ms, 32 echoes, 10 ms echo spacing, 3 cm × 2 cm field of view, 180 × 60 matrix size, 1.5 mm slice thickness, 0.5 mm gap between slices, 10 slices and 2 signal averages).

We used 1.5 mm slices and a gap of 0.5 mm between the slices instead of continuous 2 mm slices to limit the crosstalk‐related signal loss.

The values of the parameters used in diffusion‐weighted sequences were close to the values used in the Heemskerk et al^18^ study to facilitate comparison of results. We reached 12 per cent of maximal gradient amplitude using this set‐up.

MRI studies were performed at 1, 3, 7 and 14 days after femoral artery ligation in three groups of mice administered with hADSCs (hADSC group), administered with NHDF (NHDF group) and injected with PBS (control group). The number of mice evaluated per time point was equal to 4‐5 in each group. Total imaging time was 1 hour.

### 2.7 MRI data analysis

Quantitative parametric images were obtained on pixel‐by‐pixel basis with paravision 6.0 (Bruker) software.

Mean diffusivity (MD) was calculated according to the equation:

(1) $$\text{MD}=\frac{\lambda_{1}+\lambda_{2}+\lambda_{3}}{3}$$

where *λ*_1_, *λ*_2_ and *λ*_3_ denote the first, second and third eigenvalue of the diffusion tensor.

Fractional anisotropy was computed using the following equation:

(2) $$\text{FA}=\sqrt{\frac{3}{2}}\sqrt{\frac{{\left(\lambda_{1}-\text{MD}\right)}^{2}+{\left(\lambda_{2}-\text{MD}\right)}^{2}+{\left(\lambda_{3}-\text{MD}\right)}^{2}}{\sqrt{\lambda_{1}^{2}+\lambda_{2}^{2}+\lambda_{3}^{2}}}}$$

T2 maps were calculated using mono‐exponential fitting:

(3) $$S\left(\text{TE}\right)=kS_{0}\;\exp\left(-\frac{\text{TE}}{\text{T2}}\right)$$

where *k* is the proportionality constant related to signal gain or attenuation, *S*_o_ is the proton density, and TE is the echo time.

For region‐of‐interest (ROI) analysis, the parametric images were converted from the Bruker format (2dseq) to the Digital Imaging and Communications in Medicine (DICOM) files.

**Figure 1:**
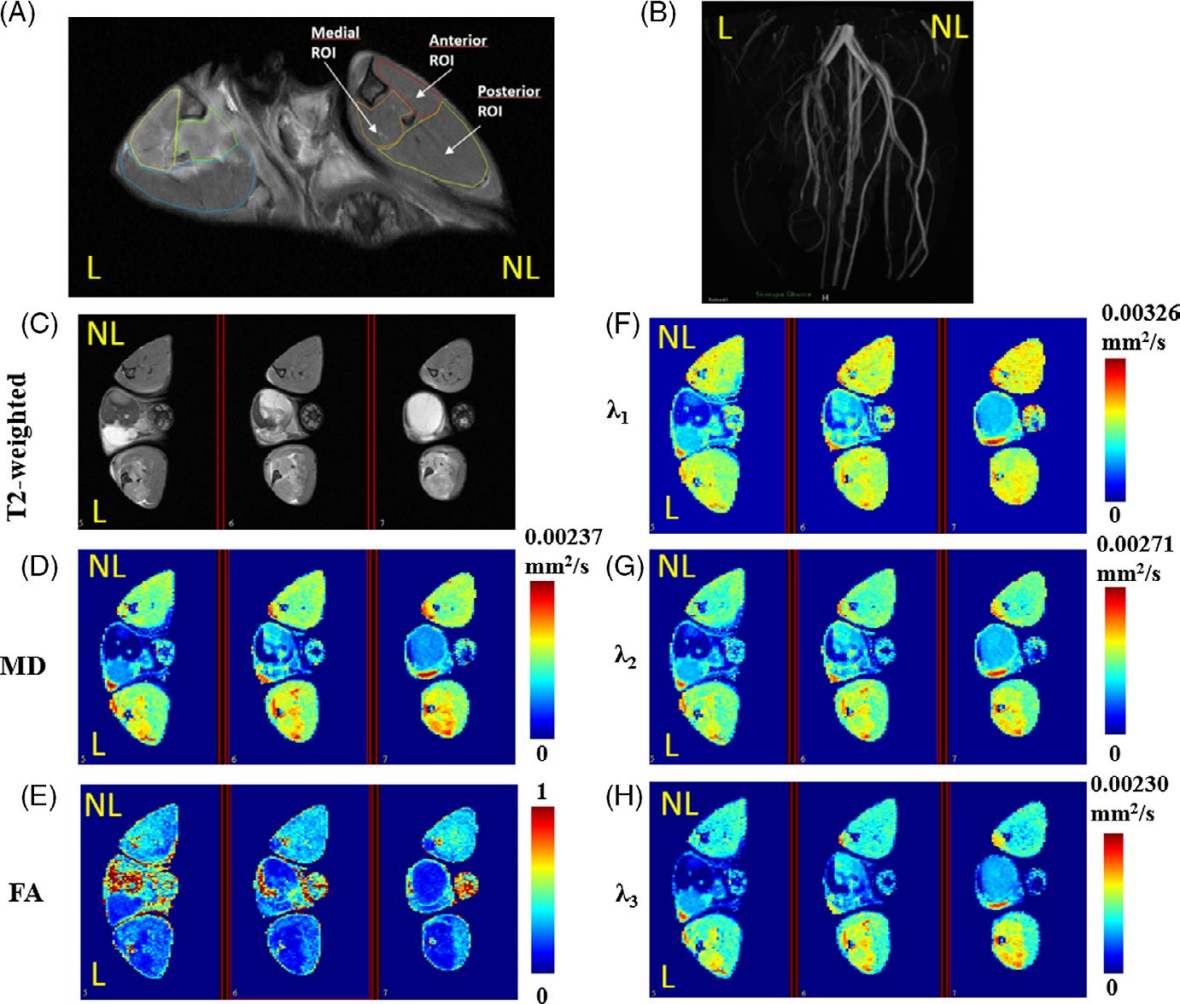
Representative T2‐weighted RARE image of the mouse hindlimbs showing the anterior, posterior and medial regions of interest (A). Representative magnetic resonance angiography image (maximum intensity projection) of the mouse hindlimbs acquired immediately after femoral artery ligation (B). Exemplary T2‐weighted RARE image (C), mean diffusivity (MD) map (D), fractional anisotropy (FA) map (E), first eigenvalue (λ_1_) map (F), second eigenvalue (*λ*_2_) map (G) and third eigenvalue (*λ*_3_) map (H) obtained from three consecutive slices of the mouse hindlimbs (control group) at day 1 after femoral artery ligation. L—ligated, NL—non‐ligated

The slices covered most of the hindlimb volume. After review of the images from a given study, 3 to 4 consecutive slices covering the calf muscle were selected for the analysis. Three 3D regions of interest were manually drawn in these sections for both ligated and non‐ligated limbs in Medical Image Processing Analysis and Visualization (mipav) software (Figure 1A). The anterior ROI includes tibialis anterior, extensor digitorum longus and peroneus, the medial one comprises flexor digitorum longus and tibialis posterior, while the posterior one gastrocnemius, soleus and plantaris. The average values of the calculated parameters in each ROI were further analysed.

### 2.8 Statistical analysis

Statistical analysis was performed with statistica software (Statsoft). The differences in the MR parameters between the ligated and non‐ligated limbs for each ROI at a given time point were assessed using the Wilcoxon signed‐rank test. The values obtained for the ligated limb were normalized to the respective values in the opposite leg to obtain the relative changes in the analysed parameters:

(4) $${\text{Parameter}}_{\text{rel}}=\frac{{\text{Parameter}}_{\text{ligated}}}{{\text{Parameter}}_{\text{non - ligated}}}$$

where Parameter denote T2, MD, FA, *λ*_1_, *λ*_2_ or *λ*_3_ values in the ligated (Parameter_ligated_) and in the non‐ligated (Parameter_non‐ligated_) hindlimbs. The Kruskal‐Wallis test followed by multiple comparisons of mean ranks was used to analyse the differences in the computed parameters (normalized to the values typical of the intact limbs) between the hADSC, NHDF and control groups.

The *P* values of < .05 were accepted as statistically significant, while those falling into range from 0.05 to 0.1 were considered to indicate trends.

Absolute values of the evaluated parameters are presented in (Figure S1).

### 2.9 Histochemical and immunohistological analyses

After 3, 7 and 14 days after surgery, mice were sacrificed and gastrocnemius muscles were dissected, frozen in liquid nitrogen and sectioned at 5 μm thickness. The capillaries were visualized by immunofluorescent staining with anti‐CD31 (Abcam). Anti‐F4/80 and anti‐CD206 antibodies (Abcam) were used to identify M2 macrophages. Respective secondary antibody conjugated with FITC or Texas Red (Vector Laboratories) was used. The slides were coverslipped using VECTASHIELD Mounting Medium with DAPI (Vector Laboratories). Imaging of the fluorescence of the stained sections was performed with the confocal microscope LSM710. Frozen sections were examined histochemically (haematoxylin/eosin staining; Sigma‐Aldrich). Analyses of the specimens were conducted using Nikon Eclipse 80i microscope (Nikon Instruments Inc).

## 3 RESULTS

### 3.1 Magnetic resonance angiography

In order to confirm the effectiveness of surgical intervention, the magnetic resonance angiogram of a mouse hindlimb vessel system was done immediately after femoral artery ligation (Figure 1B).

### 3.2 Monitoring of diffusion and relaxation properties

The exemplary T2, MD, FA, *λ*_1_, *λ*_2_ and *λ*_3_ maps from three consecutive slices of mouse hindlimbs at 3 days after the femoral artery ligation are presented in Figure 1C‐H.

#### 3.2.1 Transverse relaxation time (T2)

**Figure 2:**
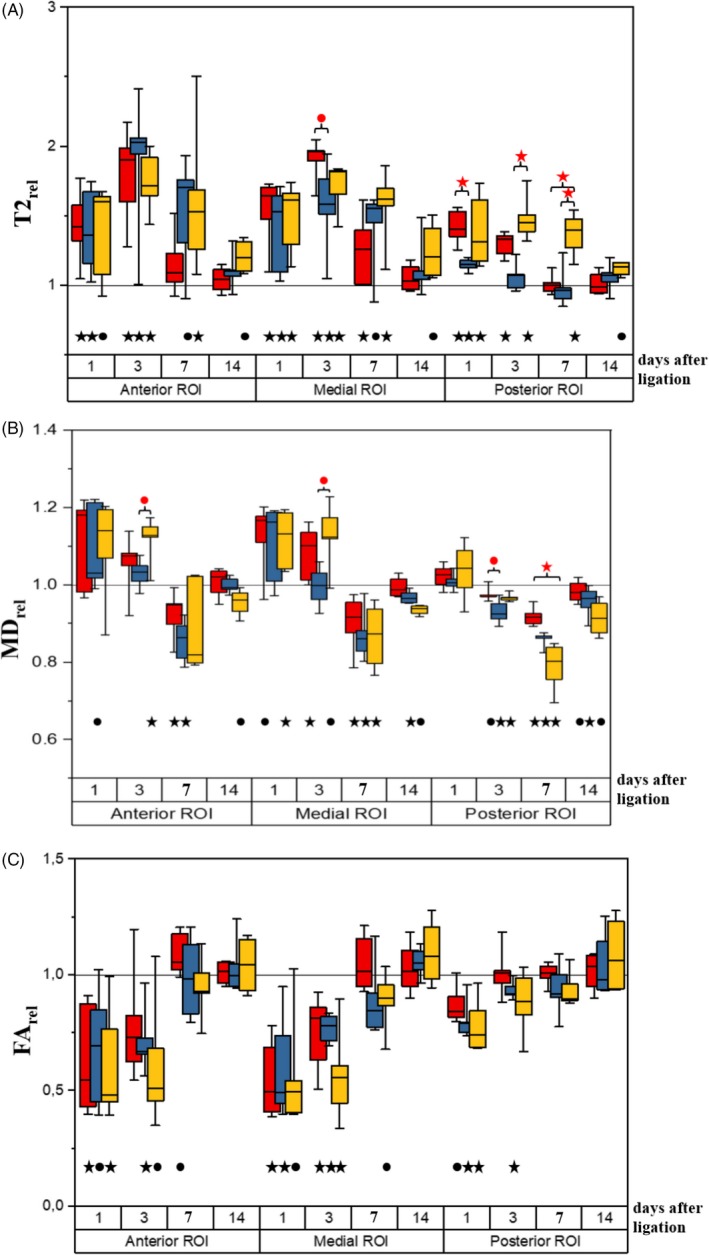
Transverse relaxation time (T2_rel_) (A), mean diffusivity (MD_rel_) (B) and fractional anisotropy (FA_rel_) (C) values in the ligated limb (normalized to the respective values in the opposite leg) at 1, 3, 7 and 14 days after femoral artery ligation for mice administered with fibroblasts (NHDF) (red), administered with human mesenchymal stromal cells derived from adipose tissue (hADSC) (yellow) and injected with PBS (control) (blue). Line—median, box—25%‐75% percentiles, whiskers—minimum and maximum. *P* values (*P* < .05—black stars, .05 < *P* < .1—black dots), obtained from the Wilcoxon signed‐rank test used for the comparison of the parameters between the ligated and non‐ligated limb. *P* values (*P* < .05—red stars, .05 < *P* < .1—red dots), obtained from the Kruskal‐Wallis test followed by multiple comparisons of mean ranks used for the comparison of the parameters between mice administered with fibroblasts (NHDF), administered with human mesenchymal stromal cells derived from adipose tissue (hADSC) and injected with PBS (control)

The results of the comparisons of the transverse relaxation times obtained for the studied groups are presented in Figure 2A.

##### Anterior and medial ROIs

Femoral artery ligation resulted in a statistically significant increase in T2 values or upward trends (relative to the non‐ligated limb) at days 1, 3 and 7 in the anterior and medial ROIs in all three groups (with the exception of T2 value in the anterior ROI at day 7 in the NHDF group, not differing from the non‐ligated limb). At day 14 for the control and NHDF groups in both ROIs, the T2 values return to the values typical for the non‐ligated limb; in case of the hADSC group, they still tend to be increased. The Kruskal‐Wallis test, however, reveals no significant T2_rel_ changes between the groups at any time point.

In the medial ROI, a trend towards increased T2_rel_ is seen at day 3 in the NHDF group in comparison with the control group.

##### Posterior ROI

In the control group, only a relatively slight T2 increase (relative to the non‐ligated limb) at day 1, followed by a subsequent normalization at later time points, is observed. However, T2 was found to be increased at days 1 and 3 in the NHDF group and during the whole evaluation period in the hADSC group (the significant changes at days 1, 3 and 7 and a trend detected at 14 days). The between‐group comparison revealed a significantly higher T2_rel_ in the hADSC group at days 3 and 7 in comparison with the control group and higher T2_rel_ in the hADSC group at day 7 as compared to the NHDF group. Additionally, it was found that at day 1, T2_rel_ is higher in the NHDF than in the control group.

#### 3.2.2 Mean diffusivity (MD)

The results of the mean diffusivity comparisons for the studied groups are shown in Figure 2B.

##### Anterior and medial ROIs

MD was significantly increased or showed an upward trend in the medial ROI (relative to the intact leg) at days 1 and 3 in the NHDF and hADSC groups. The between‐group analysis revealed that MD_rel_ at day 3 tends to be higher in the hADSC group than in the control group. In the medial ROI, the statistically significant decrease in MD (below the value for the non‐ligated leg) was noted at day 7 in all evaluated groups. Although it was still decreased at day 14 with respect to the other side in the hADSC and control groups, no significant MD_rel_ differences between the groups were found using the Kruskal‐Wallis test.

As regards the anterior ROI, a statistically significant increase in MD (relative to the non‐ligated limb) is seen at day 3 in the hADSC group. The between‐group analysis revealed that at this time point, MD_rel_ tends to be higher in this group than in the control group. MD decreases (relative to the non‐affected leg) at day 7 in the NHDF and control groups, but no statistically significant differences between the groups were found by the Kruskal‐Wallis test.

##### Posterior ROI

In all evaluated groups, a statistically significant decrease in MD or a trend towards its reduction (relative to the non‐ligated limb) was found at days 3 and 7 in this ROI. The minimum MD_rel_ value was attained for the hADSC group at day 7. However, the between‐group comparison confirms the statistically significant difference only between the hADSC and NHDF groups, whereas in case of the hADSC and control groups, the difference is statistically insignificant. Additionally, for the NHDF group, MD_rel_ tends to be increased at day 3 as compared to the control group.

#### 3.2.3 Fractional anisotropy

The results of the fractional anisotropy comparisons for the studied groups are shown in Figure 2C.

##### Anterior and medial ROIs

In the anterior and medial ROIs of all evaluated groups (with the exception of the FA change in the anterior ROI in the NHDF group, not reaching statistical significance at day 3), the FA values are markedly lower or reveal the trends towards reduction (relative to the non‐ligated limb) at days 1 and 3. Additionally, in the medial ROI of the operated limb, hADSC transplantation induced a trend towards FA reduction at day 7 relative to the other side. However, the between‐group comparisons show no statistically significant FA_rel_ changes in both ROIs.

##### Posterior ROI

While in the control group FA is decreased in the posterior ROI (relative to the non‐ligated limb) at days 1 and 3, in both other groups (hADSC and NHDF), such effect is seen only at day 1. The FA_rel_ inter‐group changes are, however, insignificant.

#### 3.2.4 First eigenvalue *λ*_1_

**Figure 3:**
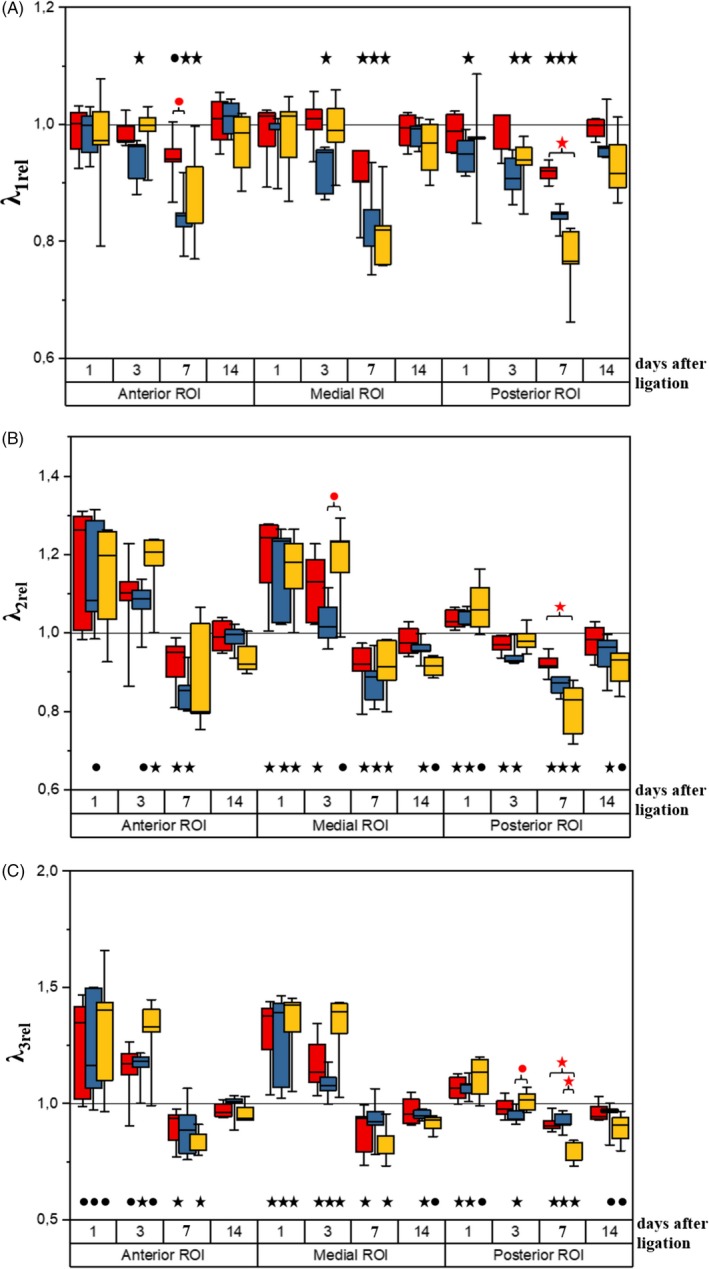
First eigenvalue (*λ*_1,rel_) (A), second eigenvalue (*λ*_2,rel_) (B) and third eigenvalue (*λ*_3rel_) (C) in the ligated limb (normalized to the respective values in the opposite leg) at 1, 3, 7 and 14 days after femoral artery ligation for mice administered with fibroblasts (NHDF) (red), administered with human mesenchymal stromal cells derived from adipose tissue (hADSC) (yellow) and injected with PBS (control) (blue). Line—median, box—25%‐75% percentiles, whiskers—minimum and maximum. *P* values (*P* < .05—black stars, .05 < *P* < .1—black dots), obtained from the Wilcoxon signed‐rank test used for the comparison of the parameters between the ligated and non‐ligated limb. *P* values (*P* < .05—red stars, .05 < *P *< .1—red dots), obtained from the Kruskal‐Wallis test followed by multiple comparisons of mean ranks used for the comparison of the parameters between mice administered with fibroblasts (NHDF), administered with human mesenchymal stromal cells derived from adipose tissue (hADSC) and injected with PBS (control)

The results of the first eigenvalue, *λ*_1_, comparisons for the studied groups are shown in Figure 3A.

##### Anterior and medial ROIs

In all studied groups, *λ*_1_ was found to be decreased or showed a downward trend in the anterior and medial ROIs located in the ligated limb (relative to the intact one) at day 7. The decrease was also apparent at day 3 in the control group. The between‐group analysis revealed a trend towards the increased *λ*_1,rel_ in the anterior ROI at day 7 in the NHDF group relative to the control group.

##### Posterior ROI

The lowest *λ*_1,rel_ in the posterior ROI was observed at day 7 in all studied groups. The between‐group comparison revealed a statistically lower *λ*_1,rel_ in the hADSC group than in the NHDF group at this time point. A statistically significant decrease in *λ*_1_ with respect to the non‐ligated limb was additionally observed at day 3 in the hADSC group, and at days 1 and 3 in the control group.

#### 3.2.5 Second eigenvalue *λ*_2_

The results of the second eigenvalue, *λ*_2_, comparisons for the studied groups are shown in Figure 3B.

##### Anterior and medial ROIs

In the medial ROI of all studied groups, the ischaemic limb *λ*_2_ values were found to be significantly increased or reveal an upward trend (relative to the non‐ligated side) at days 1 and 3 (with the exception of day 3 in the control group). In the same ROI, the between‐group analysis exposes a trend towards higher *λ*_2,rel_ in the hADSC group than in the control group at day 3.

In the anterior ROI of the control group, *λ*_2_ tends to be elevated above the level typical for the non‐ligated limb at days 1 and 3, while in the hADSC group, this parameter increases significantly at day 3. The statistically significant reductions of *λ*_2_ (relative to the non‐ligated side) in the anterior and medial ROIs at day 7 were observed in all studied groups (with the exception of the hADSC group in the anterior ROI). However, no significant *λ*_2,rel_ changes are seen between the groups in the anterior and medial ROIs at day 7 when using the Kruskal‐Wallis test.

##### Posterior ROI

In the posterior ROIs of all studied groups, the femoral artery ligation induced a statistically significant increase in *λ*_2_ or its trend towards an elevation (relative to the non‐ligated side) at day 1. However, at day 3 in the NHDF and control groups, the *λ*_2_ values are found to fall markedly below the level typical for the non‐operated limb, and at day 7, a further decrease takes place for all three groups. The between‐group comparison reveals a significantly lower *λ*_2,rel_ in the hADSC group than in the NHDF group at day 7. By day 14, *λ*_2_ returned to the values typical for the non‐operated limb only in mice injected with NHDF, but the Kruskal‐Wallis test shows no differences between the groups.

#### 3.2.6 Third eigenvalue *λ*_3_

The results of the third eigenvalue, *λ*_3_, comparisons for the studied groups are collected in Figure 3C.

##### Anterior and medial ROIs

In the anterior and medial ROIs of all evaluated groups, the statistically significant increases of *λ*_3_ or its trends towards elevation (relative to the non‐ligated limb) are observed at days 1 and 3. Moreover, the hADSC and NHDF administration leads at day 7 to a statistically significant reduction of this parameter in the operated limb as compared to its value in the healthy leg. However, the between‐groups comparisons performed for both ROIs using the Kruskal‐Wallis test reveal no significant changes in *λ*_3,rel_. While *λ*_3_ in the anterior ROI returned to the values typical of the intact leg in all groups by day 14, these values were slightly decreased in the control and hADSC groups in the medial ROI at this time point.

##### Posterior ROI

In the posterior ROI, the femoral artery ligation effect—the *λ*_3_ increase—is seen already at day 1 in all evaluated groups (relative to the non‐ligated limb). This elevation is statistically significant for the NHDF and control groups, while for the hADSC group, only a trend is observed. In the operated limb (for all studied groups), *λ*_3_ is evidently lower as compared to the other side at day 7. Of note, the between‐group analysis indicates a significant reduction of *λ*_3,rel_ in the hADSC group at day 7 as compared to the other groups. Moreover, the comparison of the hADSC group and the control one lets us notice a trend towards the *λ*_3,rel_ elevation at day 3. Although a decreasing *λ*_3_ trend in the operated limb vs the healthy limb is seen at day 14 in these groups, the Kruskal‐Wallis test does not show significant changes.

### 3.3 Summary of the differences in relaxation and diffusion properties of injured muscle between hADSC, NHDF and control groups

**Figure 4:**
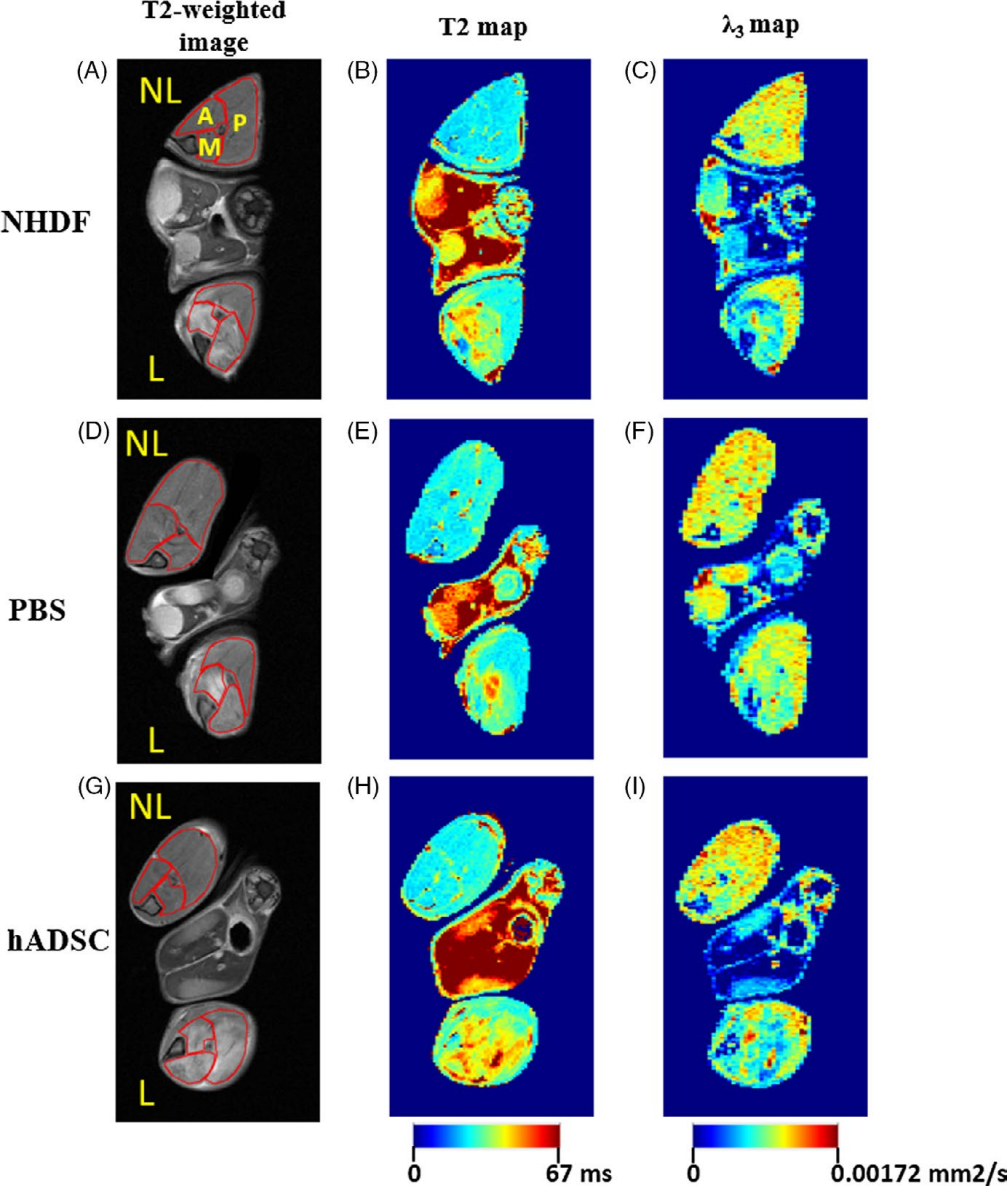
Representative T2‐weighted images (A, D, G) showing the analysed regions of interest (A—anterior, M—medial, P—posterior), transverse relaxation time (T2) maps (B, E, H) and third eigenvalue (*λ*_3_) maps (C, F, I) acquired at day 7 after femoral artery ligation in a mouse administered with fibroblasts (NHDF group) (A, B, C), injected with PBS (control) (D, E, F) and administered with human mesenchymal stromal cells derived from adipose tissue (hADSC group) (G, H, I). L—ligated, NL—non‐ligated

Summarizing, the most significant differences between the hADSC, NHDF and control groups were observed around day 3 after femoral artery ligation (increased T2_rel_ with respect to the control group) and at day 7 (increased T2_rel_ and decreased *λ*_3,rel_ in comparison with the control and NHDF groups) at the site of hADSC injection (the posterior ROI). The representative T2‐weighted images, T2 maps and *λ*_3_ maps acquired at day 7 after femoral artery ligation are presented in Figure 4.

### 3.4 Histological analysis

**Figure 5:**
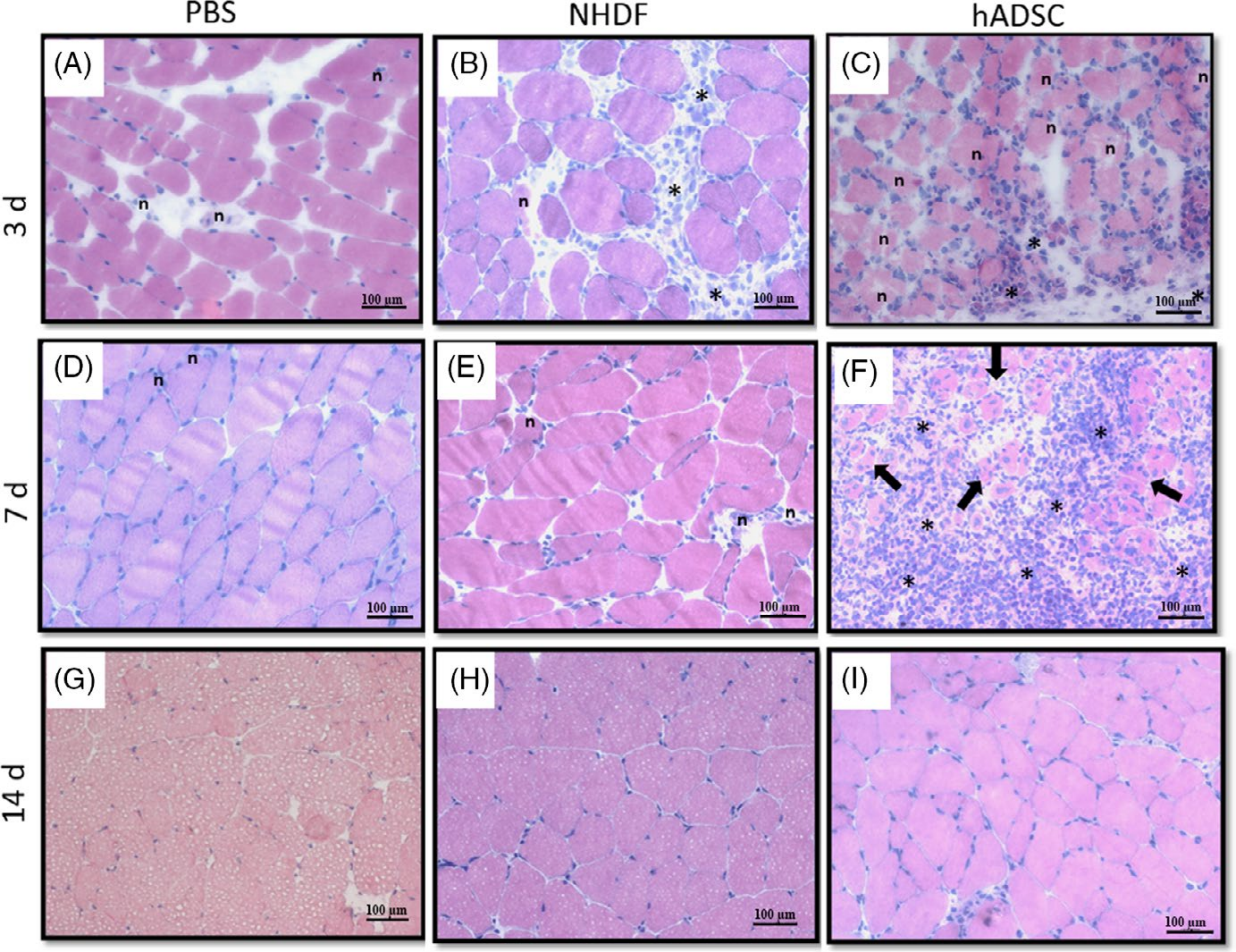
Representative images of transverse sections of gastrocnemius muscle tissues (stained by haematoxylin and eosin) obtained at 3 (A, B, C), 7 (D, E, F) and 14 (G, H, I) days after artery ligation and subsequent administration of PBS (control group), human fibroblasts (NHDF) and human mesenchymal stromal cells isolated from adipose tissue (hADSC). Infiltration of immune cells (*—asterisk) were observed at 3 (C) and 7 (F) days after hADSC administration and at 3 days (B) after NHDF administration. A few mononuclear cells were observed at 7 days after NHDF administration (E). Many necrotic muscle fibres (n) with pale cytoplasm were observed at 3 days after administration of hADSC (C), and a few necrotic muscle fibres with no nuclear staining and with irregular internal architecture were observed at 3 days after PBS^‐^ administration (A). Regenerative small muscle fibres with one or more centrally located nuclei (black arrows) were observed at 7 days after hADSC administration (F). At 14 days after hADSC administration (I), the fibres were typically polygonal and the nuclei were located peripherally, which is characteristic of normal muscle. Scale bar: 100 µm (×20 magnification)

Figure 5 shows the transverse sections of gastrocnemius muscle tissues stained by haematoxylin and eosin for the control, NHDF and hADSC groups at 3, 7 and 14 days after artery ligation. Infiltration of immune cells was observed at 3 and 7 days after hADSC injection and at 3 days after NHDF administration. A few mononuclear cells were observed at 7 days after fibroblast administration. Many necrotic muscle fibres with pale cytoplasm were observed at 3 days after administration of hADSC, and a few necrotic muscle fibres with no nuclear staining and with irregular internal architecture were observed at day 3 after PBS^−^ administration. Regenerative small muscle fibres with one or more centrally located nuclei were observed at 7 days after hADSC administration. At 14 days after hADSC administration, the fibres are typically polygonal and the nuclei are located peripherally, which is characteristic of normal muscle.

**Figure 6:**
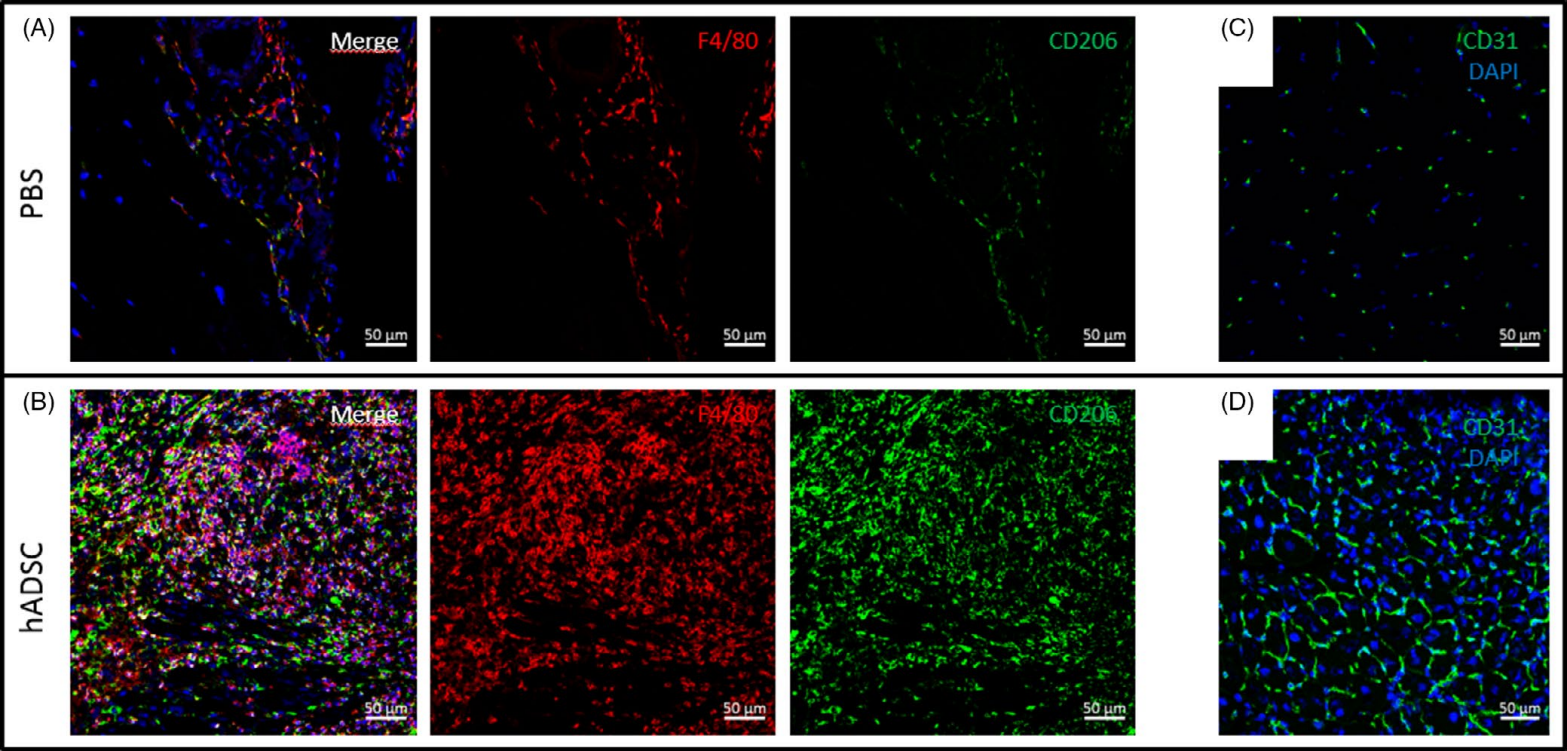
Representative images of M2 macrophages (F4/80^+^/CD206^+^) and capillaries in gastrocnemius muscle sections at 7 days after artery ligation. The macrophages were stained for F4/80 (red) and CD206 (green) in the control group (A) and in the hADSC group (B). Capillaries were visualized by immunofluorescent staining with CD31 antibody (green) in the control group (C) and in the hADSC group (D). Nuclei were stained with DAPI (blue), magnification × 20

Figure 6 (A and B) shows increased infiltration of macrophages in the hADSC group at day 7 in comparison with the control group. Almost all macrophages (F4/80^+^ cells, red) also expressed CD206 (green) indicating the M2 phenotype of these cells. The density of CD31^+^ endothelial cell‐lined capillaries was increased in muscles of mice injected with hADSC in comparison with the control group (Figure 6 C and D).

## 4 DISCUSSION

In this study, we demonstrated the effects of xenotransplantation of hADSCs on dynamics of degeneration and regeneration processes after femoral artery ligation in immunocompetent mice. We also analysed the ischaemic tissue response to human NHDF cells. These procedures triggered a cellular and antibody response—it was monitored with T2 relaxometry and DTI and verified using immunohistochemical methods. The results imply that MRI may be very useful in tracking MSC treatment effectiveness and provide valuable preclinical information for the development of xenogeneic therapies for tissue repair.

Applications of MSCs in the treatment of various disorders have been studied for almost three decades.^7^, ^27^ The obtained results indicate that their therapeutic effect is visible despite a short survival time after administration. The retention rates below 10% measured 24 hours after injection to the ischaemic heart and 2% after injection to the limb were reported.^28^, ^29^ Agrawal, et al (2014) showed that hADSCs transplanted into subcutaneous and inguinal fat pad do not persist for longer than 3 weeks (90% reduction in cells number by day 10) in immunocompromised mice, while the clearance rate for immunocompetent recipients is faster. Of note, short survival time was associated with macrophage infiltration even in immunodeficient mice.^30^ Results from our laboratory indicate that hADSCs were still present in the gastrocnemius muscles of the injured limbs at 14 days after delivery.^24^

Tissue engineers focus their attention on development of effective MSC delivery methods to increase the persistence of transplanted cells. However, it is not clear whether a longer survival time will improve the therapeutic efficacy. MSCs are believed to work through ‘touch and go’ mechanism—they trigger immunomodulatory processes early after transplantation and leave the tissue or die.^7^, ^31^ Rapid clearance of MSCs may be a rationale for transplantation of these cells across species. The survival time of these cells may be sufficiently long to initiate therapeutic processes and short enough to prevent detection of inter‐species differences by the immune system of the host.^8^

Rodent models of hindlimb ischaemia have been often used in the studies of therapeutic potential of MSCs.^4^, ^5^, ^6^ These cells were found to improve muscle repair by stimulation of formation of new vessels. A combination of laser Doppler perfusion imaging (providing information mainly about skin perfusion) and histological assessment of ischaemic tissue was used for evaluation of treatment effectiveness in the majority of studies. In the context of cellular therapy, MRI technique has been mostly used for anatomical tracking of superparamagnetic iron oxide nanoparticle–labelled cells. This method is useful in visualization of cells homing but provides limited information on their in vivo viability.^32^ Signal changes detected on T2*‐weighted images were found to represent macrophages that engulfed labelled MSCs rather than living MSCs, implicating a significant role of these phagocytes in mediating the therapeutic effects of MSCs. Another approach could rely on using MRI to non‐invasively monitor the intrinsic biophysical and biochemical muscle properties in response to administration of therapeutic cells. Madonna et al^33^ exploited magnetic resonance techniques (angiography, STIR imaging and in vivo spectroscopy) to evaluate the effect of allogenic ADSC transplantation on the muscle tissue status at 4 weeks after femoral artery ligation in mice. In our work, the modification of relaxation and diffusion properties of ischaemic muscle tissue by xenogenic transplantation was evaluated. Since many studies indicate that MSC and fibroblasts are indistinguishable from each other,^34^ we also focused on the ischaemic tissue response to human NHDF cells.

T2 relaxation time is a time constant for a decay of transverse magnetization signal during MR experiment. This decay reflects intramuscular water content and its organization. Although it is possible to observe four exponential nature of this decay in the normal muscle using specialized techniques, global T2 changes determined from the mono‐exponential fitting of the MR signal acquired using standard multiecho sequences are usually reported.^16^, ^18^, ^19^, ^20^, ^21^

Our results revealed a spatial heterogeneity of T2 alterations after femoral artery ligation in the control group, in agreement with the study of Zaccagini et al,^20^ While T2 was found to be elevated at days 1‐7 in the anterior and medial ROIs (median T2_rel_ > 1.35 at all time points, with a maximum around day 3), the posterior ROI was characterized by a slight T2 increase at day 1 only (median T2_rel_ value of 1.15). The T2 elevation observed by Zhang et al^21^ already 2 hours after ligation was explained as resulting from oedema, while the huge increases around day 3 were associated with the influx of inflammatory cells to the tissue.^18^ The T2_rel_ increase observed by us 24 hours after ligation in all ROIs was accompanied by the *λ*_2,rel_ and *λ*_3,rel_ eigenvalue elevation. While *λ*_1_ reflects diffusion along the fibre direction, the origin of the *λ*_2_ and *λ*_3_ changes is not certain. Karampinos et al^35^ suggested that these eigenvalues reflect the short and long cross‐sectional axes of the fibres. The presence of swollen and round cells during the first days after artery ligation associated with increased *λ*_2_ and *λ*_3_ seems to confirm this hypothesis.^18^ However, cell swelling is often accompanied by expansion or structural modification of extracellular space. Galbán et al^36^ supported the notion that *λ*_3_ could be a marker of the cross‐sectional diameter of the fibre, while *λ*_2_ was proposed to reflect the diffusion properties of extracellular endomysium. While the T2_rel_ values in the anterior and medial ROIs in the control group were still increased at day 7, the MD values were found to be decreased relative to the level typical for a non‐occluded limb. At day 14, both relaxation and diffusion parameters in the ligated limb were close to the values typical for the intact leg, although some indexes were still significantly changed with respect to the other side. The published results indicate that complete normalization of the diffusion and relaxation parameters should be expected within 3‐4 weeks after artery ligation.^18^, ^19^, ^20^ However, a direct comparison of our results with the reported data is difficult due to the variability in mice strains, mice age and diversity of surgical procedures of artery ligation. C57BL/6NCrl mice demonstrate quick spontaneous recovery from ischaemia after artery ligation/occlusion. Thus, the monitoring period in our study was limited to the initial 14 days after artery ligation spreading over the most dynamic changes in the muscle architecture and inflammatory status.

Transplantation of hADSCs resulted in a modification of relaxation and diffusion properties of ischaemic muscles at the site of injection (posterior ROI) as compared to those for the control and NHDF groups. The hADSC group of mice demonstrated higher T2_rel_ than the control group (at days 3‐7) and the NHDF group (at day 7). Esposito et al^15^ found a positive association between the global T2 and the number of infiltrating leucocytes during the course of muscle degeneration and regeneration after cardiotoxin injection. In our study, extensive macrophage infiltration at days 3‐7 was visible in the tissue sections collected from the posterior ROI (gastrocnemius muscle) from mice administered with hADSC.

Macrophages constitute a heterogenous group of cells known for their regulatory role in the healing processes. While M1 macrophages stimulate inflammatory response, M2 macrophages show anti‐inflammatory and pro‐angiogenic phenotype. However, recent works underscore the importance of the coordinated action of cells representing both phenotypes in efficient tissue vascularization.^37^

Although the precise mechanisms of immunomodulatory and pro‐angiogenic effects of MSCs are unknown, a growing body of data indicate that these effects are mediated by macrophages.^38^ Transplanted MSCs were shown to increase the recruitment of these phagocytes and cause a switch from M1 to M2 polarization accompanied by increased angiogenesis in the injured tissue.^39^, ^40^, ^41^ It has been speculated that this switch is related to engulfment of dead MSCs by macrophages.^31^, ^42^ Our immunohistochemical analysis confirmed the M2 phenotype of macrophages after hADSC transplantation. The results from our laboratory indicate that the crucial role in this process is played by interleukin‐6 secreted by hADSCs.^24^ This cytokine stimulates the M2 macrophages responsible for the repair of damaged muscle and formation of new blood vessels.^24^

Although the observed T2_rel_ increase in the hADSC group is probably related to the presence of inflammatory cells at days 3‐7, a possible interrelation between T2 relaxation and microcirculation was speculated on the basis of a mouse model of hindlimb ischaemia.^19^ The areas characterized morphologically as ‘early regeneration’ were described by high T2 values and high density of newly formed microvessels. Since these immature vessels are likely to be hyperpermeable, the resulting T2 elevation was interpreted as being due to fluid accumulation in the interstitial space. However, as the authors of this study point out, T2 elevation could be also influenced by inflammation.

Further insights into the relation between T2 and microcirculation were provided by Araujo et al^43^ Their biexponential analysis of the transverse magnetization decay behaviour under variable vascular filling conditions indicated that vascular space affects markedly the long component of the fit. Progressive increase in the vascular space was associated with a significant increase in global T2 values.

While the mechanism responsible for T2 increase after administration of hADSCs is complex, valuable complementary information may be gained from the analysis of diffusion properties of the ischaemic muscle. As revealed from our study, transplantation of hADSCs leads to a statistically significant decrease in *λ*_3,rel_ at the site of injection (posterior ROI) at day 7 with respect to the control and NHDF groups. In this group, the *λ*_1,rel_ and *λ*_2,rel_ values were also lowest at this time point, though a statistically significant between‐group difference appears only in relation to the NHDF group. Since the diffusion‐weighted sequence at short echo time reflects mainly intracellular space,^44^ the observed *λ*_3,rel_ decrease could be associated with the presence of immature regenerating myofibres of small diameter with centrally positioned nuclei visible in tissue sections collected from gastrocnemius muscle in the hADSC group. Of note, we did not observe histological signs of active muscle regeneration (small fibre diameter and central nucleation) in the posterior ROI of the control and NHDF groups. Slightly reduced *λ*_3rel_ (median value around 0.9) in the ischaemic posterior ROI at day 7 in these groups could be related to muscle fibre atrophy caused by diminished use of the affected limb during the first week after ligation. However, precise matching of MRI slices to histological section (not attempted in this study) is required to confirm such subtle effects.

Moon et al^5^ also observed in their nude mice undergoing hADSC transplantation (at 14 days after ligation) lower cross‐sectional areas of the fibres than in the control group, and the presence of centrally located nuclei. Such features were interpreted as the signs of increased regeneration of the tissue after hADSC transplantation. However, no infiltration of the muscle tissue with inflammatory cells after hADSC administration at this time point was mentioned.

Decreased eigenvalues of the diffusion tensor at 14 and 21 days after femoral artery ligation were observed as an effect of human endothelial progenitor cell administration into ischaemic limbs in immunosuppressed mice.^22^ Interestingly, these modifications were accompanied by enhanced infiltration of macrophages and increased angiogenesis.

In our work, diffusion imaging and relaxometry were sensitive to changes in inflammatory and regeneration muscle status after hADSC xenogenic transplantation. This underscores the potential of these techniques for testing of therapeutic protocol modifications (with respect to dose of cells, injection site, timing of administration in relation to surgery, etc.). Given the known dependency of hADSC phenotype on the initial inflammatory status of the transplantation site,^13^ it would be valuable to check the effect of hADSC injection into the anterior or medial ROIs (characterized by more intensive T2 changes caused by the artery ligation than those observed in the posterior ROI). Better understanding of the relation between biophysical properties of tissue at the time of transplantation and the treatment outcomes would facilitate finding non‐invasive biomarkers of hADSC therapy effectiveness directly translatable to human studies.

## 5 CONCLUSION

By using T2 relaxometry and DTI as well as histology, we found that injection of hADSCs into ischaemic hindlimbs in immunocompetent mice resulted in a modification of muscle degeneration and regeneration processes with respect to the control and NHDF groups. The most significant differences were observed around day 3 after femoral artery ligation (increased T2 in the hADSC group in comparison with the control group) and around day 7 (increased T2 and decreased *λ*_3_ in the hADSC group in comparison with the control and NHDF groups) at the site of hADSC injection. Histologically, it was associated with increased macrophage infiltration at days 3‐7 and the presence of small regenerating fibres in ischaemic tissue at day 7. Our results underscore the important role of macrophages in mediating the therapeutic effects of hADSCs and confirm the huge potential of magnetic resonance imaging in monitoring of cellular therapy effects.

## CONFLICT OF INTEREST

The authors declare no conflict of interest.

## AUTHOR CONTRIBUTIONS

AS, TC, MS and SS conceived and designed the study. MC, AS and ŁB contributed to the acquisition of MRI data. EP, RS, TC and MJB performed MSC isolation, femoral artery ligation and immunohistochemical analyses. AS, M, EP, TC, RS, MJB and ŁB analysed and interpreted the data. ŁK performed intraoperative material collection. AS, EP, TC, RS, MJB, MS and SS contributed to the preparation of the manuscript. All authors read and approved the final manuscript.

## Data Availability

The data that support the findings of this study are available from the corresponding author upon reasonable request.
