# Supplementary material for: Monitoring of diffusion properties and transverse relaxation time of mouse ischaemic muscle after administration of human mesenchymal stromal cells derived from adipose tissue
Source: Cell Prolif. 2019 Aug 23;52(6):e12672. doi: 10.1111/cpr.12672 (PMC6869084; doi:10.1111/cpr.12672)
Supplement: Supplementary file 1 [file CPR-52-e12672-s001.docx]

Figure S1 presents the absolute values of the evaluated diffusion and relaxation parameters in the studied groups. Kruskal-Wallis test followed by multiple comparisons of mean ranks was used for the comparison of the parameters between mice administered with fibroblasts (NHDF), human mesenchymal stromal cells derived from adipose tissue (hADSC) and injected with PBS (control). The p values of less than 0.05 were accepted as statistically significant, while those falling into range from 0.05 to 0.1 were considered to indicate the trends.

The principal findings of the study obtained based on the values measured in the ligated limb normalized to the contralateral side are also visible using the absolute values of these parameters.

Specifically:

- T2 values in the posterior ROI were found to be elevated in the hADSC group at day 3 in comparison to the control group,
- T2 values in the posterior ROI were found to be elevated in the hADSC group at day 7 in comparison to the NHDF group and an upward trend is apparent in this group relative to the control group at this time point,
- MD and λ_1_ values in the posterior ROI were found to be decreased in the hADSC group in comparison to the NHDF group at day 7,
- λ_3_ values in the posterior ROI were found to be decreased in the hADSC group in comparison to the NHDF and control groups at day 7.

The analysis of the absolute values of the parameters reveals additionally the increased MD, λ_1_, λ_2_ and λ_3_ in some ROIs in the hADSC group in comparison to the NHDF group or the control group at day 3 and an increased T2 in the medial ROI in the hADSC group relative to the NHDF group at day 7. However, these changes are not apparent after normalization of the parameters in the ligated limb to the non-ligated one.


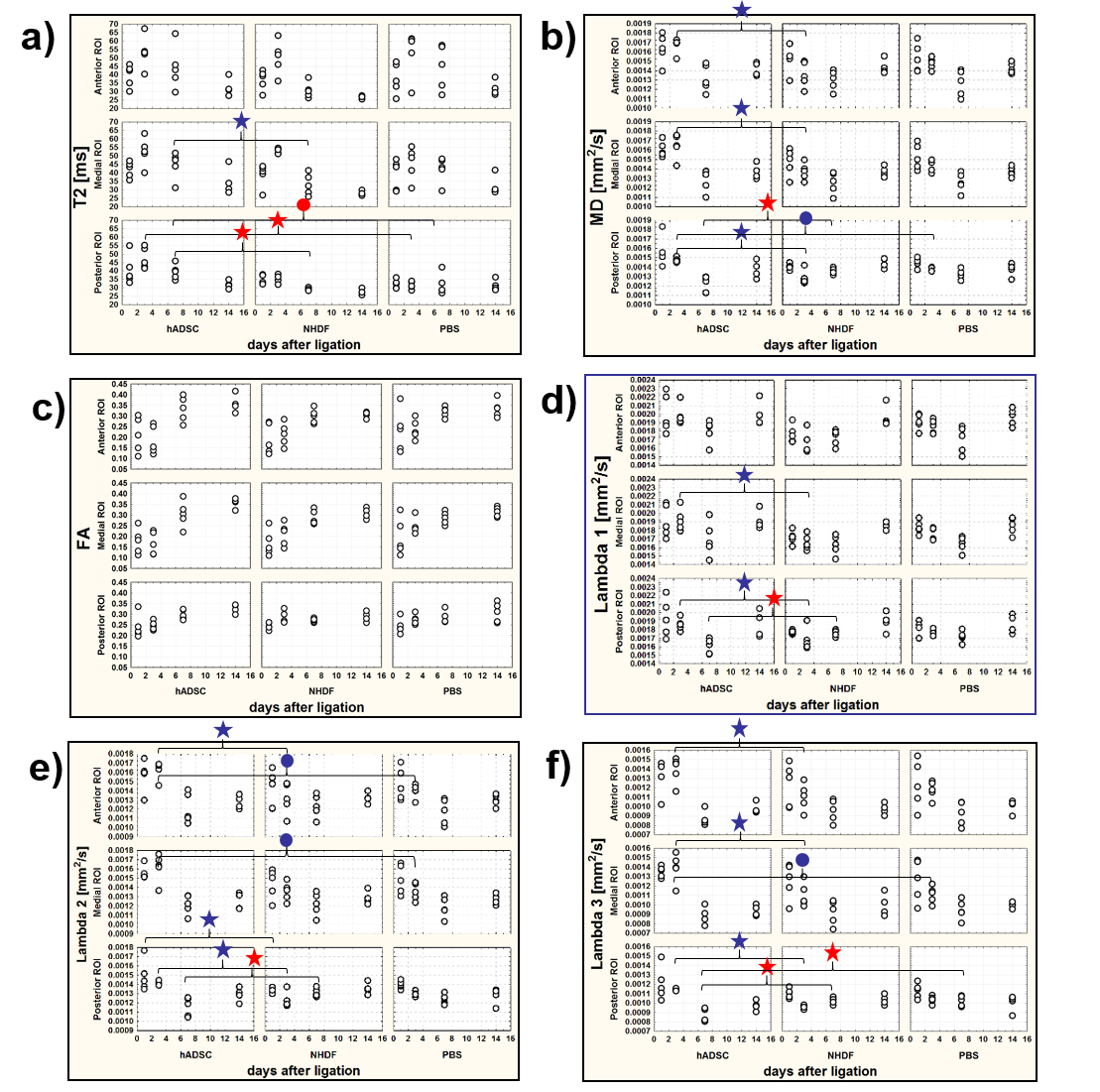


**Figure S1. Transverse relaxation time (T2) (a), mean diffusivity (MD) (b), fractional anisotropy (FA) (c), first eigenvalue (λ_1_) (d), second eigenvalue (λ_2_) (e) and third eigenvalue (λ_3_) (f) of the diffusion tensor in the ligated limb at 1, 3, 7 and 14 days after femoral artery ligation in mice administered with fibroblasts (NHDF group), human mesenchymal stromal cells derived from adipose tissue (hADSC group) and injected with PBS (control group).**

**P values (p < 0.05 - stars, 0.05 < p < 0.1 – dots), obtained from the Kruskal-Wallis test followed by multiple comparisons of mean ranks used for the comparison of the parameters between the groups.**

**The changes obtained both from the analysis of ratios (ligated leg/non-ligated limb) and from the analysis of absolute values of the parameters are marked with a red color while the remaining changes are marked with a blue color.**
